# Postoperative survival of EGFR-TKI-targeted therapy in non-small cell lung cancer patients with EGFR 19 or 21 mutations: a retrospective study

**DOI:** 10.1186/s12957-017-1251-z

**Published:** 2017-11-06

**Authors:** Wenjing Yang, Yibo Gao, Xuelian Li, Jing Zhang, Tiejun Liu, Xiaoli Feng, Hao Pan, Xiaofan Yang, Shuanghua Xie, Xiaoshuang Feng, Zhangyan Lv, Yonggang Wang, Zhaoli Chen, Jie He

**Affiliations:** 1 Department of Thoracic Surgery, National Cancer Center/Cancer Hospital, Chinese Academy of Medical Sciences & Peking Union Medical College, 17 Panjiayuan Nanli, Chaoyang District, Beijing, 100021 China; 2 Department of Epidemiology, School of Public Health, China Medical University, Shenyang, Liaoning Province China; 3 Department of Pathology, National Cancer Center/Cancer Hospital, Chinese Academy of Medical Sciences & Peking Union Medical College, Beijing, China; 4 Program Office for Cancer Screening in Urban China, National Cancer Center/Cancer Hospital, Chinese Academy of Medical Sciences & Peking Union Medical College, Beijing, China

**Keywords:** Non-small cell lung cancer, EGFR mutation subtypes, Targeted therapy, Postoperative survival

## Abstract

**Background:**

The aim of this retrospective study is to identify epidermal growth factor receptor (EGFR) mutations in non-small cell lung cancer patients and to compare the long-term postoperative outcomes in different EGFR-TKI-targeted therapy effects between the different EGFR mutation groups.

**Methods:**

A total of 2094 postoperative non-small cell lung cancer (NSCLC) patients with EGFR gene detection were collected in the Department of Pathology in the Cancer Hospital Chinese Academy of Medical Sciences from January 2003 to January 2014. Three hundred sixty-three patients were treated with EGFR tyrosine kinase inhibitor (TKI) after surgery: 184 harbored the exon 19 deletion mutation and 179 cases carried the exon 21 L858R point mutation. The end points included progression-free survival (PFS), overall survival (OS), and the response rate.

**Results:**

OS was increased in the EGFR exon 19 deletion group compared with the exon 21 L858R point mutation group (92 vs. 65 months; *P* < 0.001). But the median PFS did not differ between two groups (12 vs 14 months). The objective response rate (ORR) in 19 deletion group was increased compared with L858R mutation patients (28.35 vs. 22.73%). The disease control rate (DCR) of patients with 19 deletion benefited more from targeted therapy, compared with L858R group (93.71 vs. 84.31%, *P* = 0.014). In 19 deletion group, a high ORR and DCR were noted in patients treated with icotinib, 16 out of 18 achieved stable disease (SD), and the DCR in this population was 100%.

**Conclusions:**

EGFR subtypes could influence the postoperative survival of NSCLC patients with TKI-targeted therapy.

## Background

Non-small cell lung cancer (NSCLC) accounts for 80 to 85% of all common types of lung cancer, and NSCLC exhibits the highest morbidity and mortality rate, which is still increasing [1]. Fortunately, developments in lung cancer research over the past decade have codified major advances in the pathogenesis and management of lung cancer, especially adenocarcinoma. The identification of epidermal growth factor receptor (EGFR) mutations in NSCLC and the association between EGFR mutations and gefitinib sensitivity have changed the way lung cancer is diagnosed and treated [2]. Previous retrospective and prospective trials reported that in patients with EGFR mutations, gefitinib or erlotinib produces response rates of 70 to 80%, especially in advanced NSCLC patients with activating EGFR mutations [3, 4]. Furthermore, patients with EGFR mutations have significantly longer survival than those with wild-type EGFR when treated with EGFR tyrosine kinase inhibitors (TKIs) [4–7]. Testing for EGFR mutations is now recommended to guide patient selection for therapy involving an EGFR inhibitor and a routine postoperative examination, especially in Chinese NSCLC patients [8, 9]. However, published results have often been inconsistent and occasionally contradictory regarding the efficiency of targeted therapy as an independent treatment for NSCLC patients [10]. Most previous studies focus on the efficacy of TKI compared with chemotherapy drugs in patients with EGFR mutations, and there are also conflicting reports on whether patients with EGFR mutations are sensitive to this type of treatment [10, 11]. How to address the clinical utility and predict the benefit of taking EGFR-TKI is still unanswered.

The aim of this retrospective study is to identify EGFR mutations in NSCLC patients and to compare the long-term postoperative outcomes in different EGFR-TKI-targeted therapy effects between the different EGFR mutation groups.

## Methods

### General materials

All consecutive lung cancer subjects who underwent EGFR mutation testing in the Department of Pathology in the Cancer Hospital Chinese Academy of Medical Sciences were collected from January 2003 to January 2014. A total of 2094 postoperative NSCLC patients with EGFR gene detection were retrospectively reviewed. Then, patients meeting the following criteria for inclusion were enrolled in the present retrospective study: (i) NSCLC with a histopathological diagnosis, (ii) harboring a specific EGFR mutation (the exon 19 deletion mutation or/and the L858R point mutation), (iii) treated with EGFR-TKI until the disease progressed or toxicity was intolerable, and (iv) clinical and survival data of all patients were complete and up to date on January 1, 2015. Patients with a secondary lung tumor combined with tuberculosis, tuberculous pleural effusion, and other types of tumors or carriers of other types of EGFR mutations were excluded. In total, 363 patients aged 46 to 75 years (average age of 59.7 ± 11.6 years) were available for the present analysis. Of these patients, 184 harbored the exon 19 deletion mutation and 179 cases carried the exon 21 L858R point mutation. A total of 16 (4.41%) patients had squamous cell lung carcinoma or large cell lung cancer, whereas 347 (95.59%) patients had adenocarcinoma.

General data, including gender, age, tumor type, tumor stage, and TNM stage, and diameter of the tumor were collected as baseline information. After the subjects were enrolled, the patients were followed up every 6 months until progression; thereafter, they were observed every 6 months for survival. The primary end point of the study was progression-free survival (PFS, referred to the time span from surgery to tumor progression or death). Secondary end points included overall survival (OS) and the response rate. Four response categories included complete response (CR), partial response (PR), stable disease (SD), and progressive disease (PD) according to the effective evaluation criteria from the Response Evaluation Criteria in Solid Tumors.

### EGFR mutation testing

PCR was used for amplification and sequencing of the EGFR 19 and 21 exons. ABI Sequencing Analysis v 5.4 software (Applied Biosystems, Foster City, CA, USA) was used to analyze the sequencing results to identify EGFR mutations. A search was conducted for the following mutations: (i) EGFR exon 19 deletions and (ii) the L858R mutation (amino acid substitution at position 858 in EGFR, from a leucine to an arginine) in exon 21. EGFR 19 and 21 sites were detected in all of the cases participating in this study. TKI medicine was administered to patients with mutant EGFR, qd, or po for a duration of 4 to 8 months.

The routine follow-up procedures included a physical examination, hematological examination, computed tomography (CT) of the chest, and ultrasound of the neck and abdomen. Magnetic resonance imaging (MRI) of the head and whole-body bone scans were performed every 1 or 2 years or when recurrent disease was suspected. Routine blood examinations, heart, liver, and kidney functions, and coagulation indicators were monitored periodically. Fever, white blood cell reduction, and other complications were treated symptomatically.

### Statistical analysis

Normally distributed data are reported as the mean ± SD or as the median and interquartile range (data which deviated from the normal distribution), and the *t* test or Mann–Whitney test was used for comparisons between groups, as appropriate. Survival curves were estimated by the Kaplan–Meier product-limit method and compared with the Mantel (log-rank) test. The independent prognostic power of variances for OS and PFS was analyzed by the Cox proportional hazard method by the introduction of all covariates that were related to OS or PFS on univariate analysis or traditional confounding factors (age, sex). Hazard ratios (HR) and their 95% CIs were calculated with the estimated regression coefficients and their standard errors in the Cox regression analysis. A *P* value less than 0.05 was considered to be statistically significant. All analyses were performed using SPSS version 19.0 software (SPSS, Inc., Chicago, IL, USA).

## Results

### Patient characteristics

A total of 363 NSCLC patients were identified with activating mutations in EGFR (exon 19 deletions or exon 21 L858R point mutations). At the data cutoff point (December 31, 2014), the median follow-up period was 72 months, with a range of 5 to 134 months. Of these, 184 cases had an EGFR 19 site mutation, and 179 had an EGFR 21 site mutation. The main demographic and clinical characteristics are listed in Table 1. Patients with an exon 19 deletion were diagnosed at a younger age than exon 21 L858R point mutation patients (58.69 ± 10.19 vs. 60.87 ± 10.33). Striking differences in the distribution of the clinical stage were noted in the two EGFR mutation groups (Table 1).

**Table 1:** Baseline patient characteristics in the EGFR 19 and 21 mutation groups

	*N*	19 del (*n* = 184)	21 L858R (*n* = 179)	*P*
Age (mean ± SD)	363	58.69 ± 10.19	60.87 ± 10.33	0.044
Gender, *n* (%)				
Male	137	75 (40.76)	62 (34.64)	0.229
Female	226	109 (59.24)	117 (65.36)	
Smoking, *n* (%)				
Yes	83	47 (25.54)	36 (20.11)	0.218
No	280	137 (74.46)	143 (79.89)	
Family history, *n* (%)				
Yes	62	27 (14.67)	35 (19.55)	0.174
No	301	157 (85.79)	144 (80.45)	
Type, *n* (%)				
AD	347	175 (95.11)	172 (96.09)	0.649
Other	16	9 (4.89)	7 (3.91)	
Differentiation, *n* (%)				
High	49	23 (12.85)	26 (15.29)	0.663
Middle	246	130 (72.63)	116 (68.24)	
Low	54	26 (14.53)	28 (16.47)	
Stage, *n* (%)				0.026
I	87	52 (28.26)	35 (19.55)	
II	52	26 (14.13)	26 (14.53)	
III	168	72 (39.13)	96 (53.63)	
IV	56	34 (18.48)	22 (12.29)	
Operation, *n* (%)				0.302
Radical	288	142 (77.17)	146 (81.56)	
Palliative	75	42 (22.83)	33 (18.44)	
Adjuvant chemotherapy				0.157
Yes	285	150 (81.52)	135 (75.42)	
No	78	34 (18.48)	44 (24.58)	
Adjuvant radiation				0.395
Yes	111	60 (32.61)	51 (28.49)	
No	252	124 (67.39)	128 (71.51)	
TKI				0.886
Gefitinib	237	123 (66.85)	114 (63.69)	
Erlotinib	55	28 (15.22)	27 (15.08)	
Icotinib	58	27 (14.67)	31 (17.32)	
Others	13	6 (3.26)	7 (3.91)	

### Comparison of survival

Superior survival was observed with younger (age at diagnosis is less than 45 years) lung cancer patients (adjusted hazard ratio, 0.246; 95% confidence interval, 0.098 to 0.621) compared with patients diagnosed at over 45 years of age. Other types of NSCLC were associated with an increased hazard ratio for death compared with lung adenocarcinoma (adjusted HR 3.279, 95% CI 1.558 to 6.900). Patients with a high histological grade of differentiation had a longer median survival time than those with a low or middle differentiation (72 vs. 68 months), but survival according to the differentiation revealed no significant difference. The EGFR 19 deletion mutation is associated with prolonged survival in patients with lung cancer, with a median survival of approximately 92 months. Icotinib provides superior efficacy to gefitinib in NSCLC patients in this study, with an adjusted HR of 0.316 (95% CI, 0.137 to 0.731) (Table 2).

**Table 2:** HRs for overall survival by subgroup

	Groups	OS	*P*	cHR	95% CI		aHR	95% CI	
Age	> 45	71		1.000			1.000		
	≤ 45	–	0.022	0.349	0.142	0.859	0.246	0.098	0.621
Gender	Male	68		1.000					
	Female	92	0.400	1.087	0.895	1.320			
Smoking	No	76		1.000					
	Yes	72	0.968	1.009	0.647	1.574			
Family history	No	71		1.000					
	Yes	83	0.350	0.750	0.410	1.372			
Disease history	No	72		1.000					
	Yes	–	0.724	0.777	0.191	3.161			
Histopathologic type	AD	76		1.000			1.000		
	Non AD	53	0.027	2.260	1.097	4.659	3.279	1.558	6.900
Differentiation	High	72		1.000					
	Middle	68	0.300	1.484	0.703	3.131			
	Low	–	0.320	1.325	0.746	2.452			
Stage	I	83		1.000			1.000		
	II	68	0.209	1.507	0.795	2.857	1.455	0.751	2.282
	III	–	0.037	1.706	1.032	2.821	1.621	0.968	2.715
	IV	65	0.095	1.750	0.908	3.372	1.748	0.905	3.337
Operation	Radical	68							
	Palliative	72	0.762	1.086	0.637	1.852			
Adjuvant chemotherapy	No	76		1.000					
	Yes	67	0.688	0.904	0.554	1.477			
Adjuvant radiation	No	81		1.000					
	Yes	68	1.356	1.356	0.917	2.006			
TKI	Gefitinib	72		1.000			1.000		
	Erlotinib Hydrochloride	68	0.889	1.038	0.613	1.759	0.956	0.562	1.627
	Icotinib	–	0.022	0.379	0.165	0.871	0.316	0.137	0.731
	Others	45	0.183	1.761	0.765	4.054	1.433	0.618	3.327
EGFR mutation	19 del	92		1.000			1.000		
	21 L858R	65	0.018	1.265	1.041	1.538	1.275	1.040	1.563

**Fig. 1:**
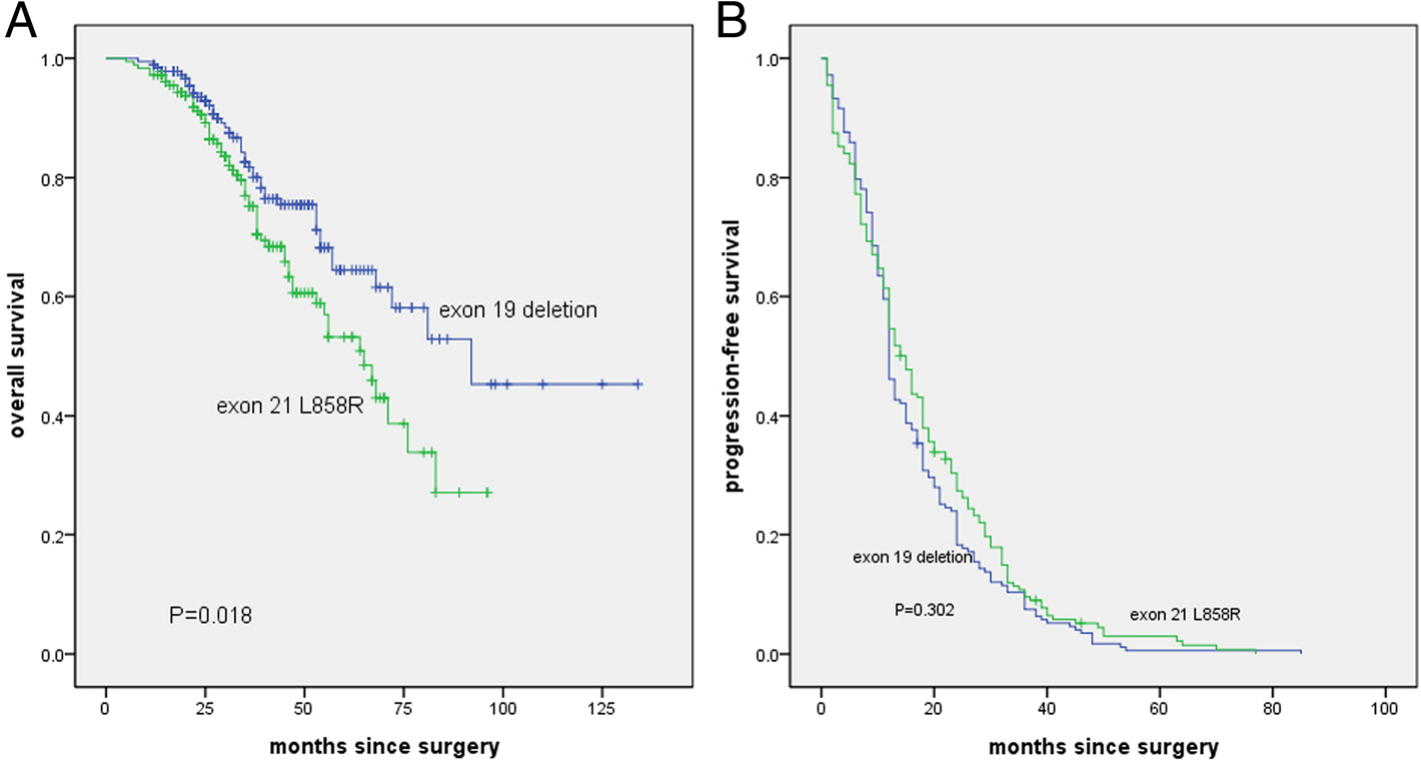
Overall survival and progression-free survival among the study patients. Kaplan–Meier curves for overall survival in the intention-to-treat population are presented (**a**). Overall survival was significantly increased in the EGFR exon 19 deletion group compared with the exon 21 L858R point mutation group (median, 92 vs. 65 months, *P* = 0.018). Kaplan–Meier curves for progression-free survival are presented for the progression-free-survival population with the two types of epidermal growth factor receptor (EGFR) mutations (**b**). The median progression-free survival did not differ significantly between patients with the EGFR exon 19 deletion and those with the L858R point mutation (12 vs 14 months, *P* = 0.302)

Overall survival was significantly increased in the EGFR exon 19 deletion group compared with the exon 21 L858R point mutation group (median, 92 vs. 65 months; hazard ratio for death, 1.265; 95% CI, 1.041 to 1.538; *P* = 0.018) (Fig. 1).

The data on progression-free survival for these patients were censored at the time of the last CT evaluation at which they did not yet have evidence of disease progression. In total, there were 177 patients with exon 19 deletions, and 169 patients with exon 21 L858R mutations had disease progression. In total, 48.48% of patients (176 patients) had disease progression within 12 months after surgery. Specifically, 96 (26.45%) patients exhibited 13 to 24 months of PFS, whereas 49 (13.50%) patients experienced 25 to 36 months of PFS. The median progression-free survival did not differ significantly between patients with the EGFR exon 19 deletion (12 months) and those with the L858R point mutation (14 months) (Table 3). Young patients were more likely to experience progression.

**Table 3:** HRs for progression-free survival by subgroup

	Groups	PFS	*P*	cHR	95% CI		aHR	95% CI	
Age	> 45	12		1.000			1.000		
	≤ 45	16	0.006	0.611	0.429	0.870	0.569	0.398	0.814
Gender	Male	12		1.000					
	Female	14	0.577	1.064	0.855	1.324			
Smoking	No	13		1.000					
	Yes	12	0.962	1.006	0.780	1.297			
Family history	No	12		1.000					
	Yes	13	0.313	0.864	0.651	1.148			
Disease history	No	13		1.000					
	Yes	10	0.158	1.578	0.838	2.972			
Histopathologic type	AD	12		1.000					
	Non AD	11	0.988	1.004	0.583	1.730			
Differentiation	High	13		1.000					
	Middle	12	0.730	0.948	0.701	1.282			
	Low	12	0.470	0.864	0.582	1.283			
Stage	I	15		1.000			1.000		
	II	12	0.209	1.507	0.795	2.857	1.039	0.729	1.481
	III	12	0.037	1.706	1.032	2.821	1.232	0.943	1.610
	IV	13	0.095	1.750	0.908	3.372	1.585	0.975	2.579
Operation	Radical	18					1.000		
	Palliative	12	0.023	1.369	1.044	1.794	1.816	1.217	2.711
Adjuvant chemotherapy	No	13		1.000					
	Yes	12	0.624	0.936	0.720	1.218			
Adjuvant radiation	No	13		1.000					
	Yes	12	0.723	1.042	0.830	1.309			
TKI	Gefitinib	13		1.000					
	Erlotinib Hydrochloride	12	0.303	1.171	0.867	1.581			
	Icotinib	13	0.177	1.225	0.912	1.646			
	Others	11	0.200	1.489	0.810	2.735			
EGFR mutation	19 del	12		1.000					
	21 L858R	14	0.302	0.946	0.851	1.052			

### Comparison of response

Owing to a lack of progression information, 83 patients (57 patients with the exon 19 deletion and 26 patients with the exon 21 L858R mutation) were excluded from the analysis of responses. The objective response rate in the exon 19 deletion group was increased compared with exon 21 L858R mutation patients (28.35 vs. 22.87%). Patients with the EGFR 19 deletion benefitted more from treatment with EGFR tyrosine kinase inhibitor (TKI) therapy (DCR 93.70 vs. 84.31%, *P* = 0.014). The differences identified were statistically significant (*P* < 0.05; Table 2). The objective response and disease control rate were similar in each TKI group in exon 21 L858R mutation patients (Table 4). In contrast, in the exon 19 deletion group, a high ORR and DCR were noted in patients treated with icotinib. In this patient group, 16 out of 18 achieved SD, and the DCR in this population was 100%.

**Table 4:** Comparison of the best response by RECIST between the two mutation groups

		CR	PR	SD	PD	ORR	DCR
19 del (*n* = 127)	Gefitinib	0	24 (27.91)	58 (67.44)	4 (4.65)	24 (27.91)	82 (95.35)
	Erlotinib	0	10 (47.62)	8 (38.10)	3 (14.29)	10 (47.62)	18 (85.71)
	Icotinib	0	2 (11.11)	16 (88.89)	0	2 (11.11)	18 (100.00)
	Others	0	0	1 (50.00)	1 (50.00)	0	1 (50.00)
	Total	0	36 (28.35)	83 (65.35)	8 (6.30)	36 (28.35)	119 (93.70)
21 L858R (*n* = 153)	Gefitinib	0	23 (23.47)	59 (60.20)	16 (16.33)	23 (23.47)	82 (83.67)
	Erlotinib	0	4 (18.18)	15 (68.18)	3 (13.64)	4 (18.18)	19 (86.36)
	Icotinib	0	8 (28.57)	17 (60.71)	3 (10.71)	8 (28.57)	25 (89.29)
	Others	0	0	3 (60.00)	2 (40.00)	0	3 (60.00)
	Total	0	35(22.87)	94 (61.44)	24 (15.69)	35 (22.87)	129 (84.31)

*CR* complete response; *PR* partial response; *SD* stable disease; *PD* progressive disease; *ORR* objective response rate, CR + PR; *DCR* disease control rate, CR + PR + SD

## Discussion

In this study, we retrospectively collected survival data of NSCLC cases to explore the impact on the postoperative survival of NSCLC patients with an EGFR mutation. The EGFR exon 19 deletion that eliminates a leucine–arginine–glutamate–alanine motif in the tyrosine kinase domain of EGFR and the thymine-to-guanine transversion that results in an arginine for leucine substitution at amino acid 858 (L858R) were the two most common EGFR mutations in NSCLC. These mutations represented 85 to 90% of EGFR mutations [12, 13]. In this population, EGFR mutations were identified in 50.33% (1054) of 2094 surgically resected non-small cell lung cancers, and 321 patients with an EGFR exon 19 deletion and 372 patients with exon 21 L858R were identified. These drug-sensitive mutations are noted in approximately 10% of Caucasian patients and up to 50% of Asian patients with NSCLC. In addition, the finding that 33.09% (95%CI, 31.09 to 35.16%) of patients harbored one of the two most common EGFR mutation genotypes was consistent with previous studies in Chinese [14] but was relatively high compared with other Asian populations (Korean population, 27.8%) [15]. In an unselected population-based cohort, 5.4% of the patients had EGFR mutation (4.3% men/6.7% women). Eighty-seven percent were activating mutations. Eight percent of adenocarcinomas and 1.9% of squamous cell carcinomas were mutated. [16] The frequency of EGFR mutations in black patients across all of these studies range from 2 to 19% [17].

Previous studies have suggested that EGFR tyrosine kinase inhibitors are highly effective against mutated-EGFR non-small cell lung cancer given that TKI may increase the binding affinities to these mutant EGFR proteins [18]. Patients with EGFR exon 19 deletions had significantly longer overall survival compared with patients with EGFR L858R after treatment with erlotinib or gefitinib [19]. Our findings confirm the benefit in OS attained with EGFR-targeted agents in Asian patients.

Why do people with exon 19 del have better survival than people with L858R? Firstly, one of the reasons was that EGFR subtype exon 19 del had higher affinities to TKIs than L858R. A study by Carey et al. [20] showed that relative binding affinity of TKI to the EGFR kinase domain was 23 times higher in the EGFR 19 deletion mutation subtype compared with EGFR L858R subtype. Another study of in vitro kinetic assay by Mulloy et al. [21] also indicated a higher affinity of TKI for exon 19 deletion than L858R mutation. Secondly, another reason of better survival with EGFR exon 19 than exon 21 L858R mutations is due to differential inhibition of downstream signals. In the study by Okabe et al. [22], TKI inhibited the phosphorylation of EGFR, Akt and Erk, to a greater degree in exon 19 deletion cells than in L858R cells. Zhu et al. [23] indicated that cell proliferation was inhibited more significantly in EGFR 19 del cells than in EGFR L858R cells in the presence of TKI. Lastly, EGFR-TKI resistant T790M mutation is more frequently encountered in pretreatment samples from patients with the L858R mutation than those with the exon 19 mutation [24]. This result suggests another possible mechanism to explain the better efficacy of EGFR-TKI in deletion 19 patients.

In this retrospective study, we also review the correlation between EGFR mutations and clinical factors that are predictive of response to TKI treatment. Patients with EGFR exon 19 deletions had significant improvements in DCR compared with patients with EGFR L858R after TKI treatment (93.70 vs. 84.31%, *P* = 0.018), but they had similar response rates (28.35 vs. 22.87%, *P* = 0.281). DCR may more accurately reflect drug efficacy and be a good candidate for one of the surrogate markers for novel types of anticancer agents in first-line and second-line or later settings [25]. Our findings strengthen this rationale. Previous randomized trials reported that the response rates to EGFR-TKIs exceed 60 to 70% in US patients [26]. However, in postoperative patients in this study, we only observed an ORR of 25%.

Gefitinib and erlotinib are first-line EGFR-TKIs that are effective at treating NSCLC. In our study, the response rates to gefitinib were 27.91 and 23.47% in the EGFR exon 19 deletion group and EGFR L858R group, respectively. The response rates to erlotinib were 47.62 and 18.18%, respectively. Significant differences in response rates determined by different mutations were observed with erlotinib. Icotinib, an oral epidermal growth factor receptor tyrosine kinase inhibitor, is a new treatment option for pretreated patients with advanced non-small cell lung cancer [26, 27]. A randomized, double-blind, phase 3 non-inferiority trial with 400 patients demonstrated that icotinib was non-inferior to gefitinib in terms of PFS but had less drug-related adverse events compared with gefitinib [27]. Our result was consisted with this study, which showed that no prolonged PFS in patients treated with icotinib were found. It showed a higher DCR and a longer OS after EGFR-TKI treatment than gefitinib and erlotinib in both exon 19 deletion and EGFR L858R group. In most studies [27, 28], icotinib have less adverse effect than other TKIs. Maybe this could improve the integrated state of patients and has better results. Moreover, the higher dose of icotinib was also well tolerated [28].

Several limitations should be taken into account when considering the results of this study. First, it was a retrospective study, not a randomized trial, and lacked a placebo control. Furthermore, all patients were postoperative patients with lung cancer who received a EGFR mutation test, and our study population may not represent the general population. Additionally, a sample size of 363 patients was sufficient to provide representative and reliable results, but it may not be sufficient for subgroup analysis. These observations warrant further confirmation in prospective studies. Further investigation is also needed to clarify the molecular mechanisms behind the varying efficacies of EGFR-TKI treatment in patients with these mutations. The efficacy of a new generation of EGFR-TKIs targeting these mutations also should be elucidated.

## Conclusions

EGFR mutations in exons 19 or 21 are correlated with clinical factors that are predictive of the response to TKI and postoperative survival in NSCLC. Patients with EGFR exon 19 deletion mutations had a longer median overall survival time compared with patients with exon 21 L858R point mutations, but the median progression-free survival time did not differ significantly. Significant variability in the DCR was noted for the different EGFR tyrosine kinase inhibitors (TKI) in patients with EGFR exon 19 deletion mutations, but not in patients with L858R point mutations. Icotinib is a new treatment option for NSCLC, which may offer survival benefits.
